# Forget me not

**DOI:** 10.7554/eLife.16597

**Published:** 2016-05-17

**Authors:** Richard GM Morris

**Affiliations:** Centre for Cognitive and Neural Systems, University of Edinburgh, Edinburgh, United KingdomR.G.M.Morris@ed.ac.uk

**Keywords:** atypical PKC, memory, PKM-zeta, PKCiota/lambda, LTP, PKMzeta, Mouse

## Abstract

An enzyme called PKM zeta may have a role in long-term memory after all.

**Related research article** Tsokas P, Hsieh C, Yao Y, Lesburguères E, Wallace EJC, Tcherepanov A, Jothianandan D, Hartley BR, Pan L, Rivard B, Farese RV, Sajan MP, Bergold PJ, Hernández AI, Cottrell JE, Shouval HZ, Fenton AA, Sacktor TC. 2016. Compensation for PKMζ in long-term potentiation and spatial long-term memory in mutant mice. *eLife* **5**:e14846. doi: 10.7554/eLife.14846

**Image** When synapses in the hippocampus are strongly stimulated, an enzyme called PKM zeta(shown in red) is produced. Images show a hippocampus before (left) and after stimulation (right); neurons are shown in green

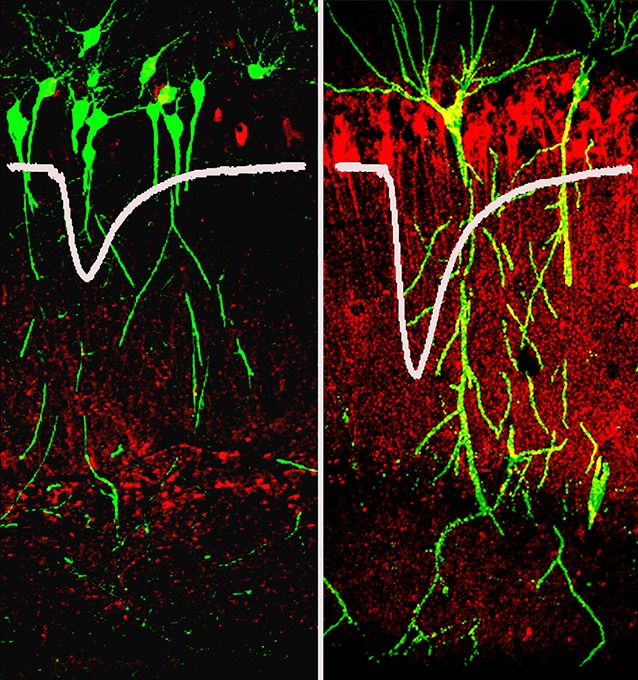

It’s great when our memory of some past event, if challenged by someone else with a different memory of it, is vindicated. Often we will simply be pleased to be right, but in some cases (such as a court of law), being right will be a matter of some importance. It must also be pleasing if you are a scientist with a theory about memory that is vindicated after being challenged by other scientists. For over 20 years, Todd Sacktor of the State University of New York (SUNY) Downstate Medical Center has been building a theory about an enzyme that he believes to be at the heart of the molecular mechanism of long-term memory. Two papers published in 2013 led many researchers to doubt this proposal but now, in eLife, Sacktor reports the results of experiments that provide new evidence that supports his theory (Tsokas et al., 2016).

The issue in question centres on how permanent memories are formed in the brain. Francis Crick wrote about memory and molecular turnover, and John Lisman developed the idea of an auto-catalytic molecule that, after an initial trigger, self-replicated its active state in the absence of further input. It is generally agreed that memory involves strengthening the synapses that connect neurons in specific parts of the brain. Sacktor has argued that this strengthening may be mediated by a lasting increase in the level of an isoform of an enzyme called PKMζ (pronounced PKM zeta) that can be rendered inherently active.

Evidence in support of this notion comes from the fact that PKMζ is known to increase the strength of synapses and, moreover, to be produced by neurons during learning. Furthermore, using a drug called ZIP (short for zeta inhibitory peptide) that inhibits the action of PKMζ prevented the formation of long-term memories (Pastalkova et al., 2006), as did the use of genetic techniques to suppress the production of PKMζ (Shema et al, 2011).

Unfortunately, in 2013, independent groups at Johns Hopkins University (Volk et al., 2013) and UCSF (Lee et al., 2013) reported that mice in which the gene for PKMζ had been knocked out were still able to form long-term memories. Moreover, they showed that while ZIP did indeed abolish memory in wild-type mice, it also abolished memories in the knock-out mice. These papers were widely discussed in the neuroscience community, with the bar-room gossip being that they had demolished Sacktor’s theory, although some researchers sought to defend his position by wondering about “redundancy and degeneracy” in the nervous system (Frankland and Josselyn, 2013). Of course, gossip should be treated with caution, as new results from Sacktor, Andre Fenton of New York University and co-workers – including Panayiotis Tsokas of SUNY as first author – suggest that a different enzyme, PKCι/λ (pronounced PKM iota lambda), is up-regulated in the absence of PKMζ and may take over some of its functions (Tsokas et al., 2016).

The researchers studied a phenomenon called long-term potentiation (LTP): this is a persistent increase in the strength of synapses and it results in the increased transmission of signals between neurons. LTP is considered to be one of the mechanisms that is responsible for learning and memory. Tsokas et al. observed that ZIP reduced late-LTP in PKMζ null mice: however, this happened because ZIP also inhibits the self-sustaining function of PKCι/λ. To explore this further Tsokas et al. created a new antisense molecule that targets the translation start site of PKMζ, predicting it would reduce late-LTP in wild-type mice but not in null mice. This prediction was upheld and validated biochemically. Additionally, while the level of PKCι/λ only increases transiently after the induction of LTP in wild-type mice (because PKMζ is doing the memory work), its level remained high throughout the experiments with the null mice. Symmetrically, a different molecule called ICAP that acts on PKCι/λ but not on PKMζ reversed late-LTP in the null mice but not in wild-type mice.

Behavioural studies using a place avoidance task by Tsokas et al. revealed that the antisense molecule disrupted long-term memory in wild-type mice but not in null mice, and that ICAP disrupted long-term memory in the null mice. A slight shadow is cast on the elegance of the story in that PKCι/λ does not perfectly compensate for lost PKMζ *in vivo.* Unexpected subtle differences were also observed in the behavioural strategies assumed by the wild-type and PKMζ null mice.

One nagging concern is whether a molecule implicated in memory retention really does need to be sustained throughout the lifetime of a memory. An alternative possibility is that it may trigger structural changes that are, in turn, mediated by other molecules (such as actin): thus, with this job done, our memory molecule can gracefully depart the scene to play upon another stage. Such structural changes could then be faithfully recycled during routine protein turnover, with these proteins being unaware, so to speak, that they are sustaining a memory.

A speculative analogy might be helpful here. Consider a spacecraft that is orbiting the earth before it is sent to the moon. For a brief period, the engines are activated, the rocket speeds up, and the spacecraft escapes earth’s gravity. It is on its way to the moon. The engines are then stopped and the rocket keeps going. Should we look for ‘molecules’ that sustain its motion towards the moon, akin to maintaining a memory as in Sacktor’s argument? Newton’s first law of motion tells us that the rocket will keep moving through space at the same velocity without help from anything else. By analogy, the molecules that make it possible for memories to be retained over long periods of time could, like the engines on a rocket, be activated only transiently. For now, the star billing for PKMζ seems to be vindicated, but time will tell whether this is sustained or transient.

## References

[bib1] Frankland PW, Josselyn SA (2013). Memory and the single molecule. Nature.

[bib2] Lee AM, Kanter BR, Wang D, Lim JP, Zou ME, Qiu C, McMahon T, Dadgar J, Fischbach-Weiss SC, Messing RO (2013). Prkcz null mice show normal learning and memory. Nature.

[bib3] Pastalkova E, Serrano P, Pinkhasova D, Wallace E, Fenton AA, Sacktor TC (2006). Storage of spatial information by the maintenance mechanism of LTP. Science.

[bib4] Shema R, Haramati S, Ron S, Hazvi S, Chen A, Sacktor TC, Dudai Y (2011). Enhancement of consolidated long-term memory by overexpression of protein kinase M in the neocortex. Science.

[bib5] Tsokas P, Hsieh C, Yao Y, Lesburgueres E, Wallace EJC, Tcherepanov A, Jothianandan D, Hartley BR, Pan L, Rivard B, Farese RV, Sajan MP, Bergold PJ, Hernandez AI, Cottrell JE, Shouval HZ, Fenton AA, Sacktor TC (2016). Compensation for pkmζ in long-term potentiation and spatiallong-term memory in mutant mice. eLife.

[bib6] Volk LJ, Bachman JL, Johnson R, Yu Y, Huganir RL (2013). PKM-ζ is not required for hippocampal synaptic plasticity, learning and memory. Nature.

